# Supplementary material for: The iron–sulphur cluster in human DNA2 is required for all biochemical activities of DNA2
Source: Commun Biol. 2020 Jun 23;3:322. doi: 10.1038/s42003-020-1048-4 (PMC7311471; doi:10.1038/s42003-020-1048-4)
Supplement: Supplementary file 1 — Supplementary Information [file 42003_2020_1048_MOESM1_ESM.pdf]

# Supplementary Information

# The iron-sulphur cluster in human DNA2 is required for all biochemical activities of DNA2

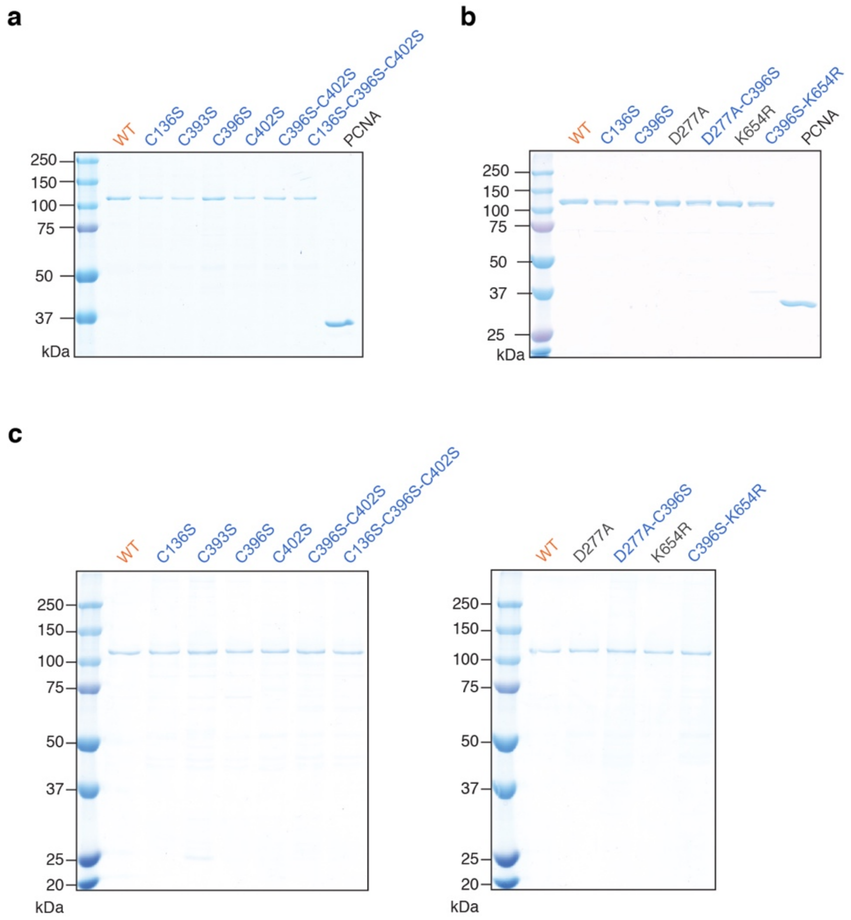


**Supplementary Fig. 1** Characterisation of the FeS cluster-deficient DNA2 variants. **a** Purified DNA2 variants from one representative radioactive iron-55 incorporation assay (quantified in Fig. 1b) were analysed by SDS-PAGE and InstantBlue staining. Protein levels were taken into account to quantify iron incorporation. **b** Purified DNA2 variants as in a (quantified in Fig. 1c). **c** Purified DNA2 variants used in biochemical assays, analysed by SDS-PAGE and InstantBlue staining.

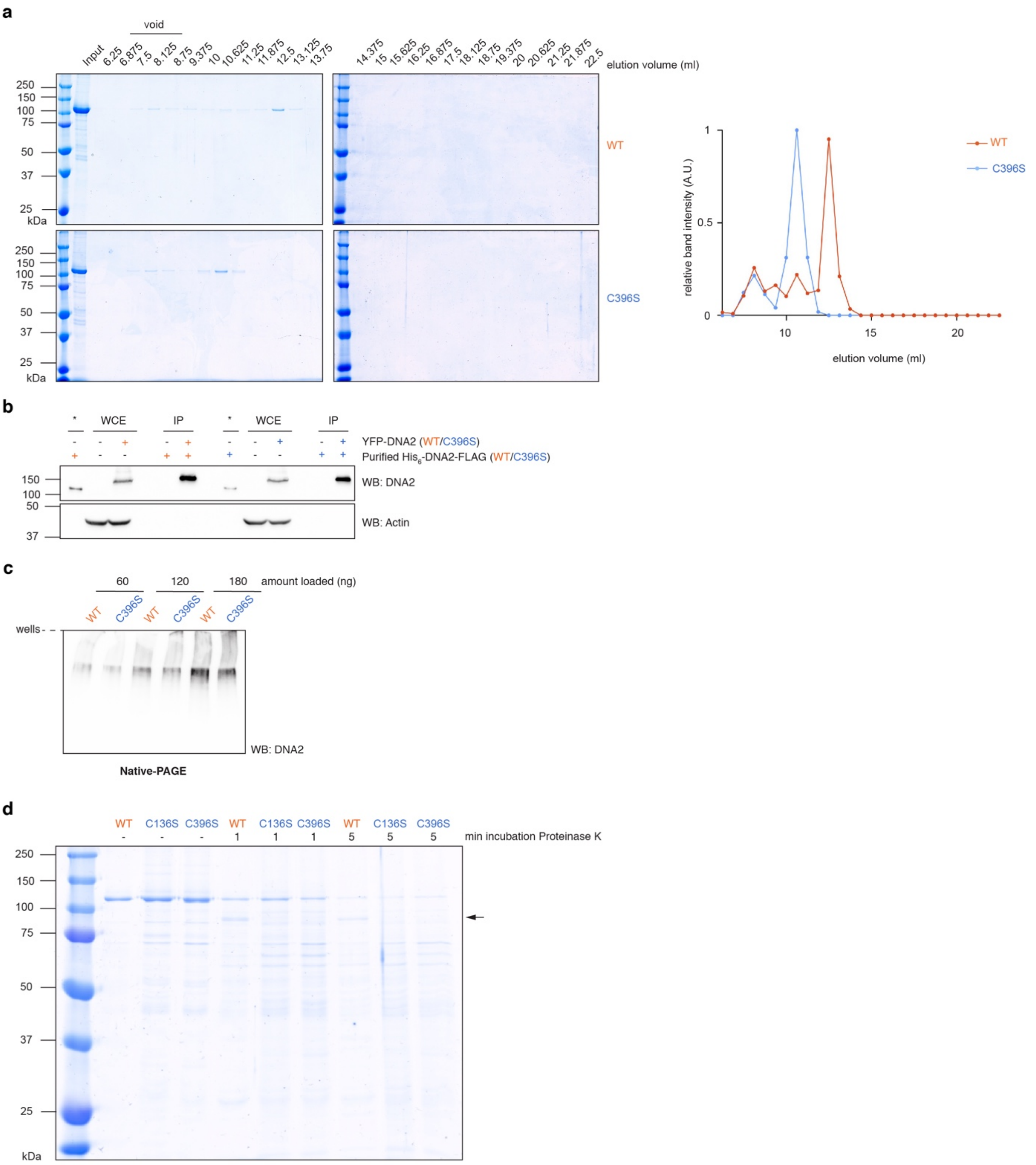


**Supplementary Fig. 2** FeS cluster loss induces a conformational change. **a** InstantBlue-stained SDS-PAGE gel of size exclusion chromatography experiment with wild-type DNA2 and the C396S variant, and quantification of the relative band intensities of the SDS-PAGE gels. **b** YFP-DNA2 (WT/C396S) over-expressed in HEK293T cells was immunoprecipitated, and co-immunoprecipitation with purified $His_6$-DNA2-FLAG (WT/C396S) was assessed by SDS-PAGE and Western blotting. Asterisk represents purified $His_6$-DNA2-FLAG alone. **c** Purified wild-type DNA2 and DNA2 C396S, analysed by Native-PAGE and Western blotting. **d** Purified DNA2 wild-type, C136S and C396S were incubated with Proteinase K for 1 and 5 min. The reactions were stopped and analysed by SDS-PAGE and InstantBlue staining. The position of the proteolytic product unique to digested wild-type DNA2 is indicated. Complete SDS-PAGE of Fig. 2d.

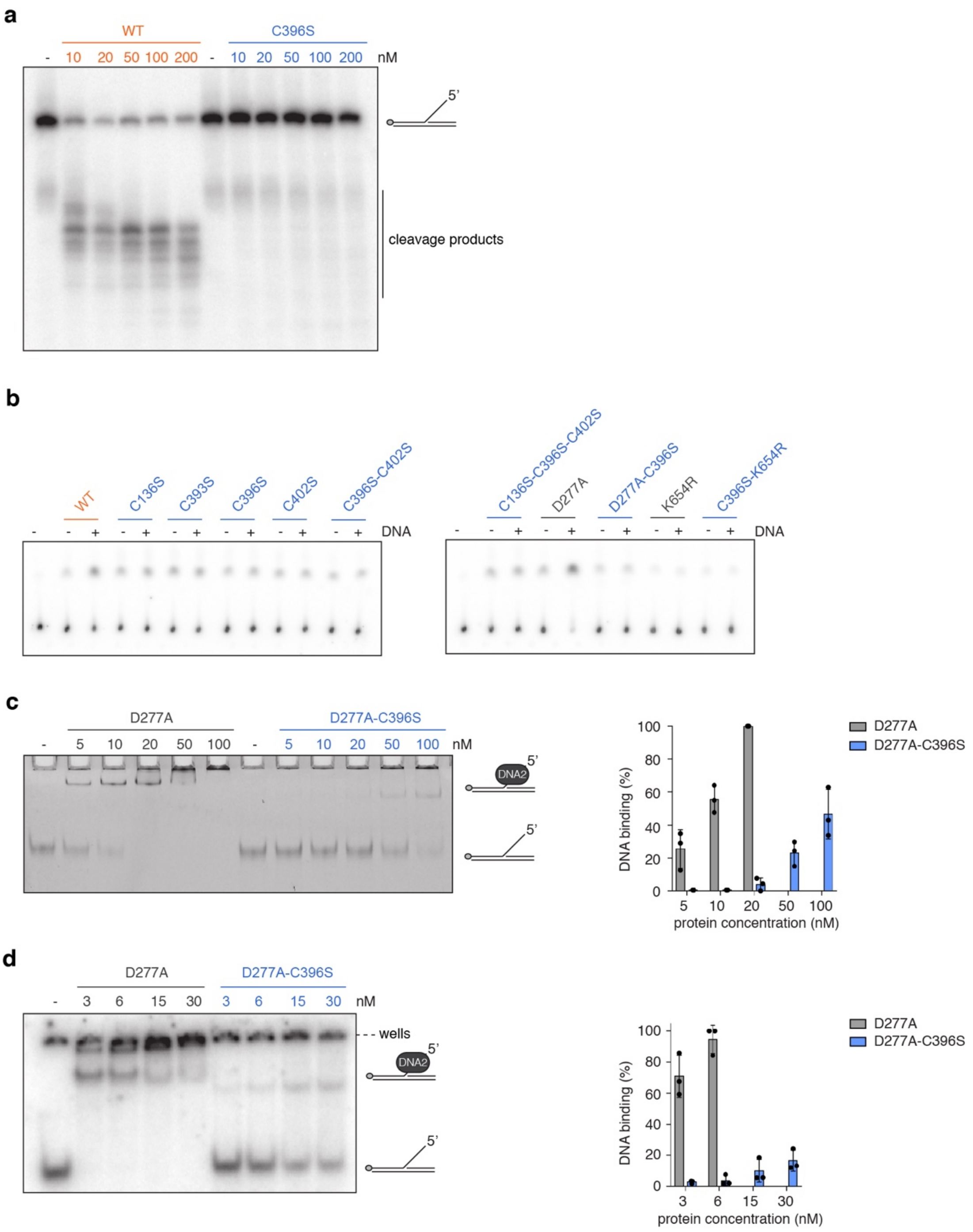


**Supplementary Fig. 3** The FeS cluster is important for DNA2's nuclease, helicase and ATPase activities. **a** Representative nuclease assay using a 5′ flap DNA substrate ($^{32}$P-labelled) at 1 nM final concentration and increasing amounts of DNA2 wild-type and C396S. **b** Representative raw image for ATPase assays quantified in Fig. 4c. **c** Representative EMSA of DNA2/DNA complexes using a 5′ flap DNA substrate (FAM-labelled) at 4 nM final concentration and increasing amounts of DNA2 D277A and D277A-C396S. Quantification of DNA binding expressed in % (n = 3 independent experiments; error bars, SD). **d** Representative EMSA of DNA2/DNA complexes using a Y-structure DNA substrate ($^{32}$P-labelled) at 1 nM final concentration and increasing amounts of DNA2 D277A and D277A-C396S. Quantification of DNA binding expressed in % (n = 3 independent experiments; error bars, SD).

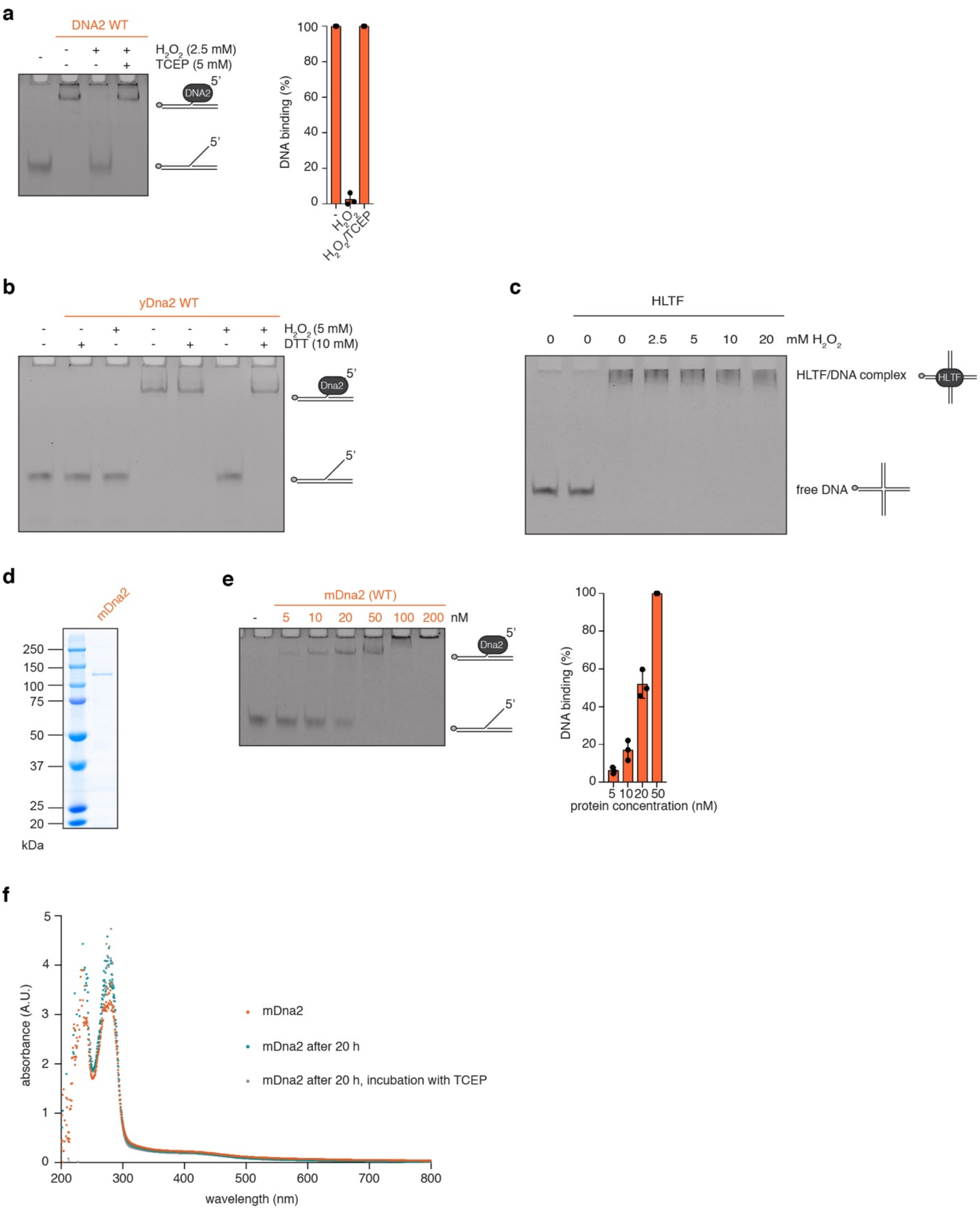


**Supplementary Fig. 4** Oxidation and reduction of DNA2 modulates DNA binding. **a** Representative EMSA of DNA2/DNA complexes with pre-incubation with 2.5 mM $H_2O_2$. 5 mM TCEP was added after the $H_2O_2$ treatment. Final protein concentration is 50 nM and final 5´ flap DNA substrate concentration is 4 nM. Quantification of DNA binding expressed in % (n = 3 independent experiments; error bars, SD). **b** Representative EMSA of yeast Dna2/DNA complexes upon pre-incubation with $H_2O_2$. DTT was added after the $H_2O_2$ treatment. Final protein concentration is 182 nM and final 5´ flap DNA substrate concentration is 5 nM. **c** Representative EMSA of human HLTF/DNA complexes upon

pre-incubation with $H_2O_2$. Final protein concentration is 13 nM and final 4-way junction DNA substrate concentration is 5 nM. **d** Purified mDna2 used in biochemical assays and UV-vis spectroscopy, analysed by SDS-PAGE and InstantBlue staining. **e** Representative EMSA of mDna2/DNA complexes using a 5′ flap DNA substrate (FAM-labelled) at 4 nM final concentration and increasing amounts of mDna2. Quantification of DNA binding expressed in % (n = 3 independent experiments; error bars, SD). **f** UV-vis spectroscopy (complete spectra) with mDna2 at 25 µM, as-purified (orange), after 20 h of air exposure (teal), and after 20 h of air exposure followed by TCEP treatment (grey).

---

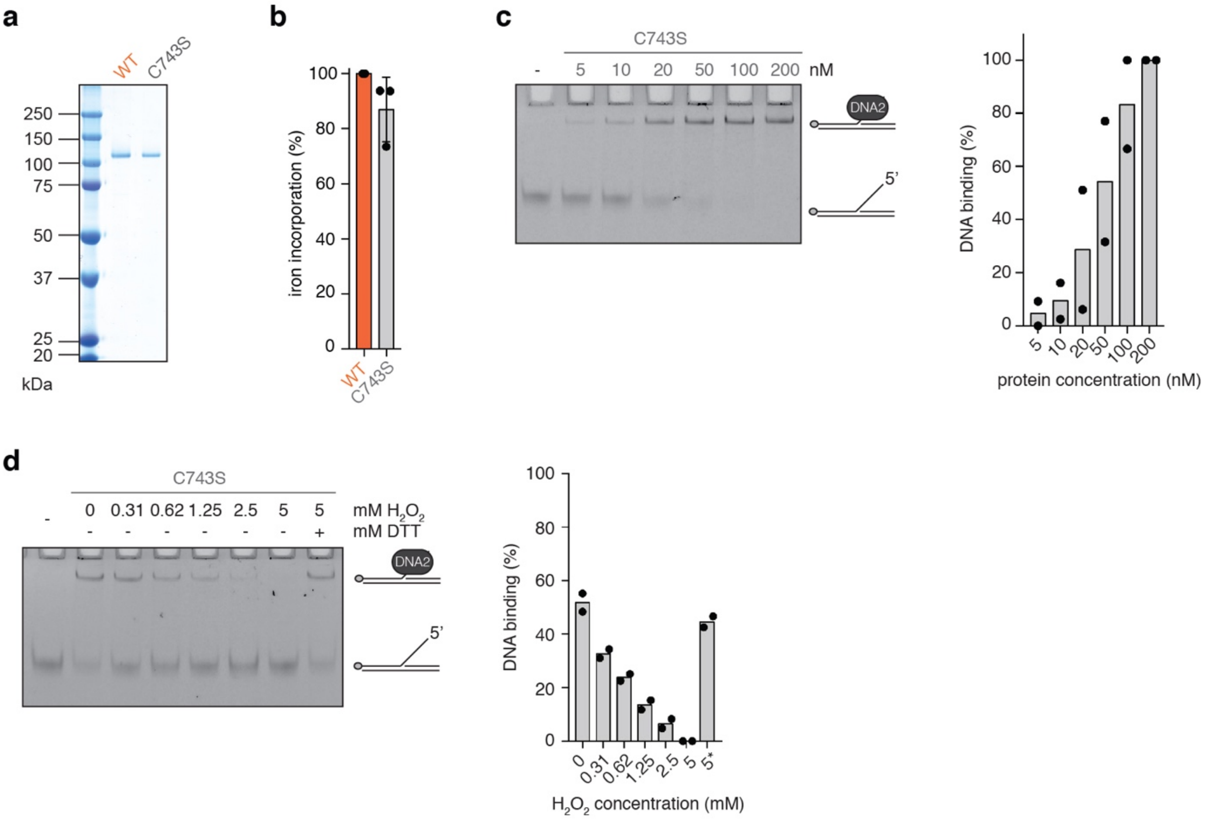


**Supplementary Fig. 5** DNA2 C743S remains sensitive to oxidation. **a** Purified DNA2 C743S used in biochemical assays, analysed by SDS-PAGE and InstantBlue staining. **b** Radioactive iron-55 incorporation in wild-type DNA2 and the C743S variant, measured by liquid scintillation counting. Data are expressed relative to wild-type, which is set to 100% (n = 3 independent experiments; error bars, SD). **c** Representative EMSA of protein/DNA complexes using a 5′ flap DNA substrate (FAM-labelled) at 4 nM final concentration and increasing amounts of DNA2 C743S. Quantification of DNA binding expressed in % (n = 2 independent experiments). **d** Representative EMSA of DNA2/DNA complexes upon pre-incubation with increasing amounts of $H_2O_2$. 5 mM DTT was added after the $H_2O_2$ treatment. Final protein concentration is 100 nM and final 5′ flap DNA substrate concentration is 4 nM. Quantification of DNA binding expressed in %. Asterisk indicates addition of DTT (n = 2 independent experiments).

**1d**

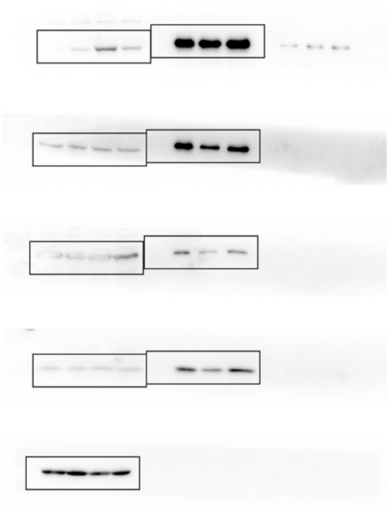

**1e**

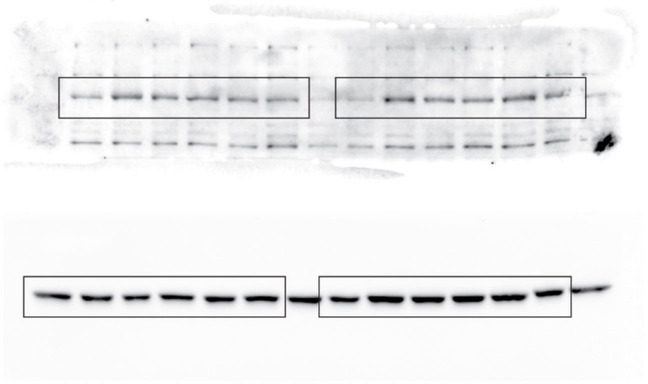

**2a**

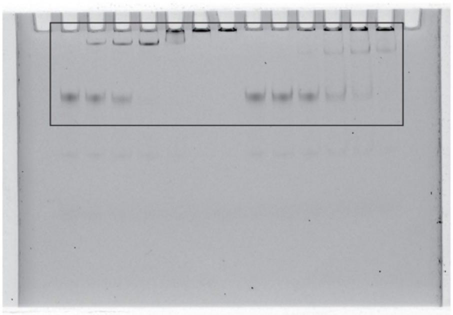

**2b**

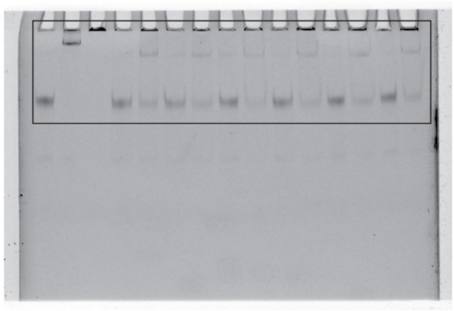

**3a**

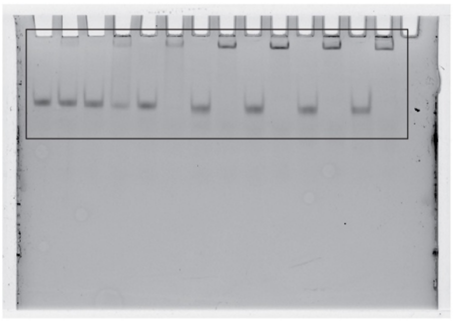

**3b**

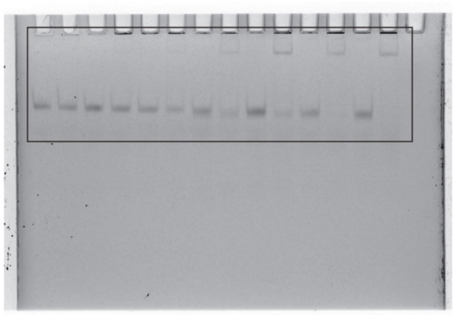

**Supplementary Fig. 6 (Part 1)** Uncropped blot and gel images. The black rectangles mark the final cropped parts, as seen in the main figures.

4a

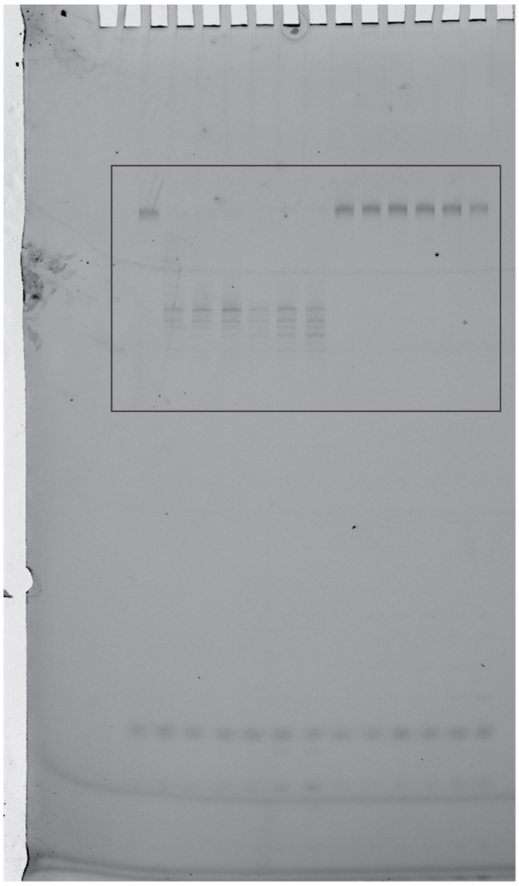

4b

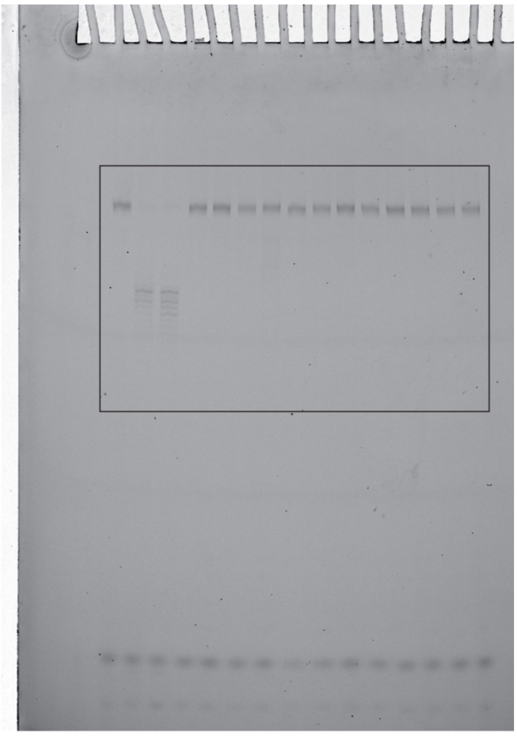

4d

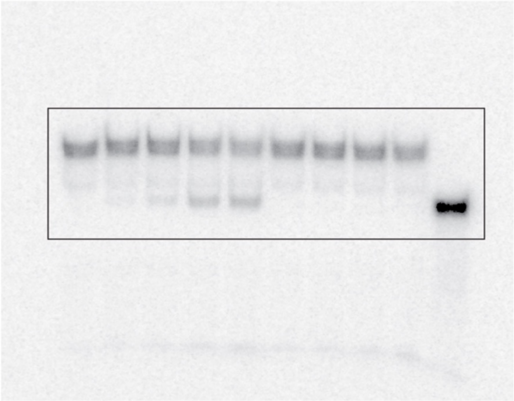

**Supplementary Fig. 6 (Part 2)** Uncropped blot and gel images. The black rectangles mark the final cropped parts, as seen in the main figures.

5a

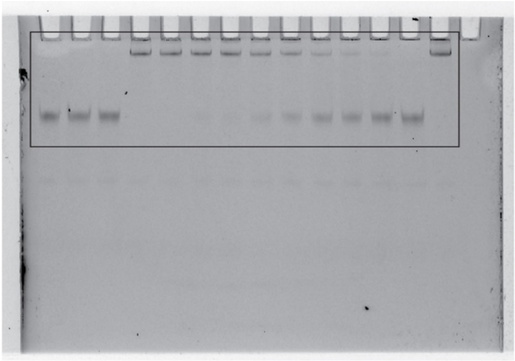

5b

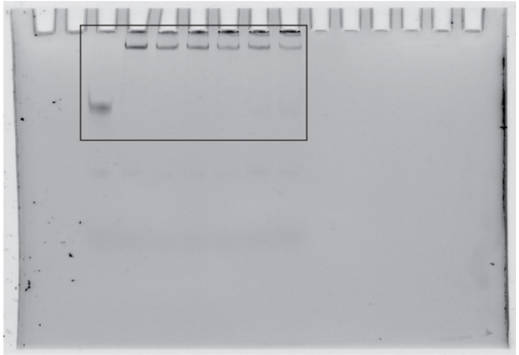

5c

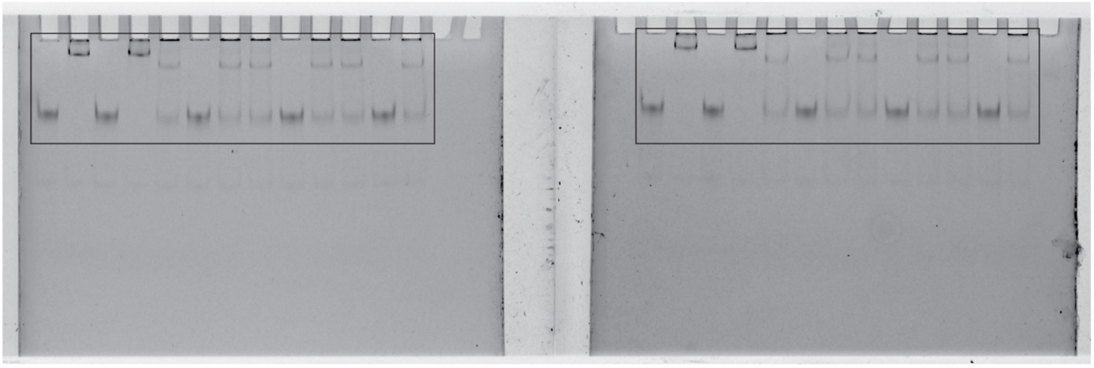

5d

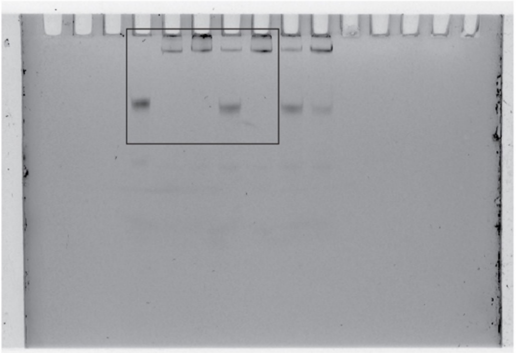

5e

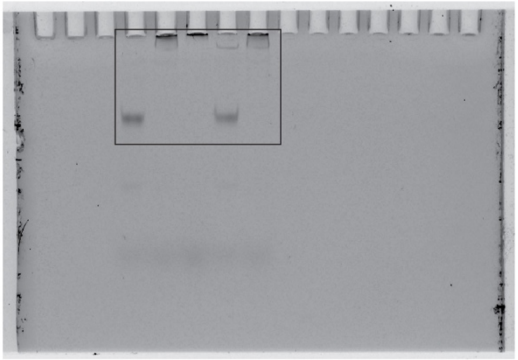

**Supplementary Fig. 6 (Part 3)** Uncropped blot and gel images. The black rectangles mark the final cropped parts, as seen in the main figures.

**Supplementary Table 1. Oligonucleotide sequences.**

| Name | Sequence 5´-3´ | Length (nucleotides) |
|---|---|---|
| **X01** | GACGCTGCCGAATTCTACCAGTGCCTTGCTAGGACATCTTTGCC CACCTGCAGGTTCACCC | 61 |
| **X02** | TGGGTGAACCTGCAGGTGGGCAAAGATGTCCATCTGTTGTAATC GTCAAGCTTTATGCCGTT | 62 |
| **X03** | GAACGGCATAAAGCTTGACGATTACAACAGATCATGGAGCTGT CTAGAGGATCCGACTATCGA | 63 |
| **X04** | ATCGATAGTCGGATCCTCTAGACAGCTCCATGTAGCAAGGCACT GGTAGAATTCGGCAGCGT | 62 |
| **X02.1/2** | TGGGTGAACCTGCAGGTGGGCAAAGATGTCC | 31 |
| **sLM2** | ACGCTGCCGAATTCTACCAGTGCCTTGCTACATGGAGCTGTC | 42 |
| **sLM3** | ACGCTGCCGAATTCTACCAGTGCCTTGCTACATGGAGCTGTCTA GAG | 47 |
| **sLM4** | ACGCTGCCGAATTCTACCAGTGCCTTGCTACATGGAGCTGTCTA GAGGATCC | 52 |
| **sLM5** | ACGCTGCCGAATTCTACCAGTGCCTTGCTACATGGAG | 37 |
| **sLM7** | ACGCTGCCGAATTCTACCAGTGCCTTGCTACA | 32 |
| **sLM9** | ACGCTGCCGAATTCTACCAGTG | 22 |
| **sLM10** | ACGCTGCCGAATTCTACCAGTGCCTTG | 27 |
| **X12-3HJ3** | GAGATCTATCTGGTGCCTTCTGACAGTGAATGGGTAACGAATCG TAATAGTCTCTAGACAGCATGTCCTAGCAATGTAATCGTCTATG ACGTC | 93 |
| **X12-3TOPL** | GACGTCATAGACGATTACATTGCTAGGACATGCTGTCTAGAGAC TATCGCGACTTACGTTCCATCGCTAGGTTATTTTTTTTTTTTTTT TTT | 93 |

**Supplementary Table 2. FAM-labelled DNA substrates.**

| DNA substrate | Labelled nucleotide | Labelled end | Unlabelled nucleotide #1 | Unlabelled nucleotide #2 | Unlabelled nucleotide #3 |
|---|---|---|---|---|---|
| 32 nt 5´-flap | X04 | 3´ | X01 | X02 1/2 | - |
| 10 nt 5´-overhang | X04 | 3´ | sLM4 | - | - |
| 15 nt 5´-overhang | X04 | 3´ | sLM3 | - | - |
| 20 nt 5´-overhang | X04 | 3´ | sLM2 | - | - |
| 25 nt 5´-overhang | X04 | 3´ | sLM5 | - | - |
| 30 nt 5´-overhang | X04 | 3´ | sLM7 | - | - |
| 35 nt 5´-overhang | X04 | 3´ | sLM10 | - | - |
| 40 nt 5´-overhang | X04 | 3´ | sLM9 | - | - |
| 32 nt Y-structure | X04 | 3´ | X01 | - | - |
| 4-way junction | X01 | 5´ | X02 | X03 | X04 |

**Supplementary Table 3. Radioactively labelled DNA substrates.**

| DNA substrate | Labelled nucleotide | Labelled end | Unlabelled nucleotide #1 | Unlabelled nucleotide #2 | Unlabelled nucleotide #3 |
|---|---|---|---|---|---|
| 32 nt 5´-flap | X04 | 3´ | X01 | X02 1/2 | - |
| 32 nt Y-structure | X04 | 3´ | X01 | - | - |
| 45 nt large Y-structure | X12-3HJ3 | 3´ | X12-3TOPL | - | - |
