# Supplementary material for: The iron–sulphur cluster in human DNA2 is required for all biochemical activities of DNA2
Source: Commun Biol. 2020 Jun 23;3:322. doi: 10.1038/s42003-020-1048-4 (PMC7311471; doi:10.1038/s42003-020-1048-4)
Supplement: Supplementary file 2 — Description of Additional Supplementary Files [file 42003_2020_1048_MOESM2_ESM.pdf]

Description of Additional Supplementary Files

File Name: **Supplementary Data 1**
Description: Source data. Raw data values for all graphs of the manuscript
