# Supplementary material for: The iron–sulphur cluster in human DNA2 is required for all biochemical activities of DNA2
Source: Commun Biol. 2020 Jun 23;3:322. doi: 10.1038/s42003-020-1048-4 (PMC7311471; doi:10.1038/s42003-020-1048-4)
Supplement: Supplementary file 4 — Reporting Summary [file 42003_2020_1048_MOESM4_ESM.pdf]

nature research

Corresponding author(s): Laura Mariotti
Kerstin Gari

Last updated by author(s): May 11, 2020

# Reporting Summary

Nature Research wishes to improve the reproducibility of the work that we publish. This form provides structure for consistency and transparency in reporting. For further information on Nature Research policies, see Authors & Referees and the Editorial Policy Checklist.

## Statistics

For all statistical analyses, confirm that the following items are present in the figure legend, table legend, main text, or Methods section.

| n/a | Confirmed | |
|---|---|---|
| ☐ | ☒ | The exact sample size ($n$) for each experimental group/condition, given as a discrete number and unit of measurement |
| ☐ | ☒ | A statement on whether measurements were taken from distinct samples or whether the same sample was measured repeatedly |
| ☐ | ☒ | The statistical test(s) used AND whether they are one- or two-sided<br>*Only common tests should be described solely by name; describe more complex techniques in the Methods section.* |
| ☒ | ☐ | A description of all covariates tested |
| ☒ | ☐ | A description of any assumptions or corrections, such as tests of normality and adjustment for multiple comparisons |
| ☐ | ☒ | A full description of the statistical parameters including central tendency (e.g. means) or other basic estimates (e.g. regression coefficient) AND variation (e.g. standard deviation) or associated estimates of uncertainty (e.g. confidence intervals) |
| ☒ | ☐ | For null hypothesis testing, the test statistic (e.g. $F$, $t$, $r$) with confidence intervals, effect sizes, degrees of freedom and $P$ value noted<br>*Give P values as exact values whenever suitable.* |
| ☒ | ☐ | For Bayesian analysis, information on the choice of priors and Markov chain Monte Carlo settings |
| ☒ | ☐ | For hierarchical and complex designs, identification of the appropriate level for tests and full reporting of outcomes |
| ☒ | ☐ | Estimates of effect sizes (e.g. Cohen's $d$, Pearson's $r$), indicating how they were calculated |

*Our web collection on statistics for biologists contains articles on many of the points above.*

## Software and code

Policy information about availability of computer code

| | |
|---|---|
| Data collection | Gels were imaged with a Typhoon FLA9500 laser scanner (GE Healthcare) |
| Data analysis | Gels were quantified using Image J (version 2.0.0-rc-43/1.52c) |

For manuscripts utilizing custom algorithms or software that are central to the research but not yet described in published literature, software must be made available to editors/reviewers. We strongly encourage code deposition in a community repository (e.g. GitHub). See the Nature Research guidelines for submitting code & software for further information.

## Data

Policy information about availability of data

All manuscripts must include a data availability statement. This statement should provide the following information, where applicable:

- Accession codes, unique identifiers, or web links for publicly available datasets
- A list of figures that have associated raw data
- A description of any restrictions on data availability

Source data are available as Supplementary Data 1. All other data are available from the corresponding authors upon reasonable request.

# Field-specific reporting

Please select the one below that is the best fit for your research. If you are not sure, read the appropriate sections before making your selection.

☒ Life sciences ☐ Behavioural & social sciences ☐ Ecological, evolutionary & environmental sciences

For a reference copy of the document with all sections, see nature.com/documents/nr-reporting-summary-flat.pdf

# Life sciences study design

All studies must disclose on these points even when the disclosure is negative.

| | |
|---|---|
| Sample size | No statistical methods were used to predetermine sample size. |
| Data exclusions | No data were excluded from the analyses. |
| Replication | Biochemical assays were repeated three times (unless stated otherwise). Consistent results could be obtained in all instances. |
| Randomization | No studies were conducted that required randomization. |
| Blinding | Data were not acquired and analysed blindly because it is not a common procedure for the biochemical assays employed in this study. |

# Reporting for specific materials, systems and methods

We require information from authors about some types of materials, experimental systems and methods used in many studies. Here, indicate whether each material, system or method listed is relevant to your study. If you are not sure if a list item applies to your research, read the appropriate section before selecting a response.

## Materials & experimental systems

| n/a | Involved in the study | |
|---|---|---|
| ☐ | ☒ | Antibodies |
| ☐ | ☒ | Eukaryotic cell lines |
| ☒ | ☐ | Palaeontology |
| ☒ | ☐ | Animals and other organisms |
| ☒ | ☐ | Human research participants |
| ☒ | ☐ | Clinical data |

## Methods

| n/a | Involved in the study | |
|---|---|---|
| ☒ | ☐ | ChIP-seq |
| ☒ | ☐ | Flow cytometry |
| ☒ | ☐ | MRI-based neuroimaging |

## Antibodies

| | |
|---|---|
| Antibodies used | The following primary antibodies were used for Western blotting: β-actin-horseradish peroxidase (HRP) (C4, sc-47778; Santa Cruz Biotechnology), DNA2 (ab96488; Abcam), CIAO1 (ab83088; Abcam), MIP18/ FAM96B (20108-1-AP; Proteintech), MMS19 (16015-1-AP; Proteintech). Secondary antibodies were anti-Mouse-HRP (NA931; Amersham) and anti-Rabbit-HRP (NA934; Amersham). |
| Validation | All antibodies used were validated previously by the providers. |

## Eukaryotic cell lines

Policy information about cell lines

| | |
|---|---|
| Cell line source(s) | HeLa Flp-In T-REx cells: gift from Stephen S. Taylor<br>HEK293T: ATCC® CRL-3216™<br>Sf9 insect cells: Invitrogen |
| Authentication | None of the cell lines were authenticated. |
| Mycoplasma contamination | Cell lines were regularly tested negative for mycoplasma contamination. |
| Commonly misidentified lines (See ICLAC register) | No commonly misidentified cell lines were used. |
